# Recovery of Walking Function After ACL Reconstruction of the Knee Joint: A Non-Randomized Study and Mixed Cross-Sectional Comparison of Postoperative Time Groups

**DOI:** 10.3390/jcm15135077

**Published:** 2026-06-29

**Authors:** Dmitry Skvortsov, Alexander Akhpashev, Aleksey Prizov, Andrey Timonin, Valery Zaharov, Alexey Gulyakovich, Anatoly Vostrikov

**Affiliations:** 1 Research and Clinical Centre, Moscow 115682, Russia; 2 Center for Brain and Neurotechnology, Moscow 117513, Russia; 3 Department of Orthopedics and Traumatology, Peoples Friendship University of Russia (RUDN University), Moscow 117198, Russia; 4 Federal Research Center for Nutrition, Biotechnology and Food Safety, Moscow 109240, Russia

**Keywords:** knee joint, anterior cruciate ligament, gait analysis, surface electromyography, muscle inhibition, postoperative recovery, long-term medical rehabilitation

## Abstract

**Background/Objectives**: Previous studies have measured a limited number of biomechanical parameters during medical rehabilitation of an anterior cruciate ligament (ACL) rupture. This study aimed to quantitatively assess changes in gait biomechanics, knee function, and lower-extremity muscle activity during after ACL reconstruction. **Methods**: The study included 32 patients after arthroscopic ACL reconstruction. The patients were divided into three groups based on postoperative time points: 0.5 year (12 men), 1 year (7), and over 1 year (9). Gait analysis at both self-selected and fast speeds was performed using an inertial system. Statistical analysis was performed using rank models and full-factorial orthogonal designs. **Results**: After 0.5 year, the timing of the gait cycle at self-selected speed was within the control group’s range and showed no significant asymmetry. With increasing speed, a decrease in knee joint range of motion was observed in the 0.5 year and 1-year groups, without achieving a full physiological increase in range of motion at long-term follow-up. Multivariate analysis revealed the greatest biomechanical imbalance during fast walking at one year and a phase-dependent effect of time after surgery, speed, and limb status on kinematics and EMG, particularly in the quadriceps. **Conclusions**: Basic temporal gait parameters during self-selected walking were within the control range by 0.5 year, but load-dependent knee kinematic and EMG abnormalities persisted. The knee joint’s response to increased loads remained impaired for at least one year. The persistence of phase-specific compensatory changes in kinematics and muscle activity at later stages can be assessed using exercise testing.

## 1. Introduction

Anterior cruciate ligament (ACL) injury is one of the most common injuries to the ligaments of the knee joint (KJ). It leads to long-term limitations of physical activity, reduced quality of life, and an increased risk of post-traumatic arthritis. Epidemiological studies show that in the general population, the incidence of primary ACL ruptures is approximately a few dozen cases per 100,000 population. In contrast, in athletes, especially in team sports, these rates are significantly higher [1,2,3]. Several meta-analyses and registry studies confirm the highest incidence in young, physically active patients, including athletes, and that, in recent years, there has been an increase in ACL ruptures in adolescents [4,5]. The number of ACL reconstructions worldwide is estimated at hundreds of thousands of procedures per year and is expected to continue to increase [6]. Given the high prevalence of this injury and the fact that a significant proportion of patients develop radiographic signs of KJ arthritis within 10–15 years after the rupture, restoring knee function after ACL injury is a relevant medical and social issue.

ACL reconstruction is currently the standard surgical treatment. It is one of the most common arthroscopic knee surgeries; however, even with anatomically correct technique and without complications, full restoration of musculoskeletal function and return to sports at the previous level are not guaranteed [7,8,9].

Biomechanical studies show that, even with a subjectively successful outcome of ACL reconstruction, changes in KJ kinematics and kinetics during walking may persist compared with those of healthy individuals [10,11]. A three-level meta-analysis of more than 800 patients after ACL reconstruction showed that the most consistent difference from controls was a decrease in the peak KJ extension angle during the stance phase. In contrast, differences in peak flexion angle, external moments, step length, and gait speed were less clear [12]. Recovery of muscle function, particularly quadriceps femoris (QF) strength and neuromuscular control, is a key factor in functional outcome after ACL reconstruction. One year after surgery, QF strength on the operated side remained reduced on average, reaching approximately 80–85% of that on the contralateral limb, while flexor strength deficits were less pronounced [13,14,15,16]. Arthrogenic Muscle Inhibition (AMI), a reflex decrease in central muscle activation associated with altered afferentation from articular mechanoreceptors, is considered a key mechanism of persistent muscle function deficit [17]. Experimental studies have shown that intra-articular effusion in the KJ not only causes spinal inhibition but also decreases corticomotor excitability, confirming the multilevel nature of AMI [18]. The effectiveness of therapy aimed at reducing AMI has not been sufficiently studied and requires further systematization [19,20].

This study aims to evaluate the recovery time course of gait biomechanics, KJ function, and lower extremity muscle function in patients 6 months, 1 year, and more than 1 year after ACL reconstruction.

The hypothesis of the study is that within one year after ACL reconstruction the biomechanics of walking is not fully restored. In this study, by restoration of walking function, we mean compliance with biomechanical normative parameters of walking at an arbitrary speed.

## 2. Materials and Methods

### 2.1. Study Design

The study was a non-randomized, interventional, mixed cross-sectional comparison of postoperative time groups in patients after ACL reconstruction.

### 2.2. Patients

The study included 32 patients who had undergone ACL reconstruction (primary arthroscopic ACL reconstruction performed using autografts from the hamstring or peroneus longus muscles). Based on the time elapsed since ACL reconstruction, the subjects were divided into three groups: 0.5 years, 1 year, and more than 1 year. These are three independent postoperative groups of patients, examined at different periods of time after surgery. Patients were admitted for examination from two different clinics, where they underwent ACL reconstruction using the same technique, but by different surgeons. Patient recruitment for the study was conducted from 2024 to 2026. The 0.5- and 1-year groups were examined at the specified time points after reconstruction ±10 days. The more-than-1-year group was examined at various time points, with a mean time point of 3 years and 2 months after reconstruction. The key data for the patient groups are presented in Table 1.

*Patient inclusion criteria* for the study included a history of ACL reconstruction without complications and patient compliance with the postoperative regimen and standard medical rehabilitation according to the appropriate stage. Patients were aged 18 to 60 years and provided written informed consent to participate in the study. No other musculoskeletal injuries or conditions that alter gait function were present.

*Exclusion criteria* included patient refusal to participate at any stage; missed follow-up examinations for more than 10 days; missed gait biomechanics assessments for more than 10 days; and the occurrence of complications or injuries affecting gait during the study period.

The study included 34 healthy adult controls, with a mean age of 29.8 ± 7.9 years and a mean BMI of 20.6 ± 1.8 kg/m^2^.

This study was conducted in accordance with the ethical principles outlined in the Declaration of Helsinki. Prior to participation in this study, all subjects signed written informed consent forms approved by the Independent Interdisciplinary Committee for Ethical Review of Clinical Research (№10/18-07-24, issued on 18 July 2024).

### 2.3. Gait Assessment

Gait biomechanics was assessed using a Steadys system (Neurosoft, Ivanovo, Russia). Seven inertial measurement units (IMUs) were used. The IMUs were attached to the sacrum and, on both legs, to the lateral middle third of the thigh, lateral lower leg above the ankle, and on the bridge of each foot. Each IMU sensor had two independent EMG recording channels. Electromyography (EMG) was recorded from the main flexor and extensor muscles: tibialis anterior (TA), both gastrocnemius (GM) muscles, quadriceps femoris (QF), and hamstrings (HM). EMG was recorded using disposable surface Mederen electrodes (Tel Aviv-Jaffa, Israel).

The IMUs sent the captured data to a computer via Wi-Fi. The kinematic and temporal data were collected at 200 Hz. The EMG channels collected native data at 2000 Hz. Subsequent EMG processing included a high-pass filter with a cutoff frequency of 5 Hz. Smoothing of the EMG profile after its rectification was performed with a constant component of 200 ms. The notch filter (50 Hz) was not used since the sensor operated autonomously from its own battery. The neural network for detecting gait cycles was trained on navigation data [21]. Data capture was completed after reaching 30 gait cycles. The software of the SteadysPlus-2.1.0.0 identified gait cycles and calculated gait-cycle parameters, including EMG profiles of muscles and joint movement goniograms normalized to the gait cycle. We analyzed the electrical activity profile of each muscle during the gait cycle (μV), based on smoothed and rectified EMG profiles that were normalized to the gait cycle, as were the goniograms. All linear envelopes of EMG or goniogram were averaged with respect to the gait cycles. Each final EMG profile or goniogram included 200 individual values per gait cycle.

**Figure 1:**
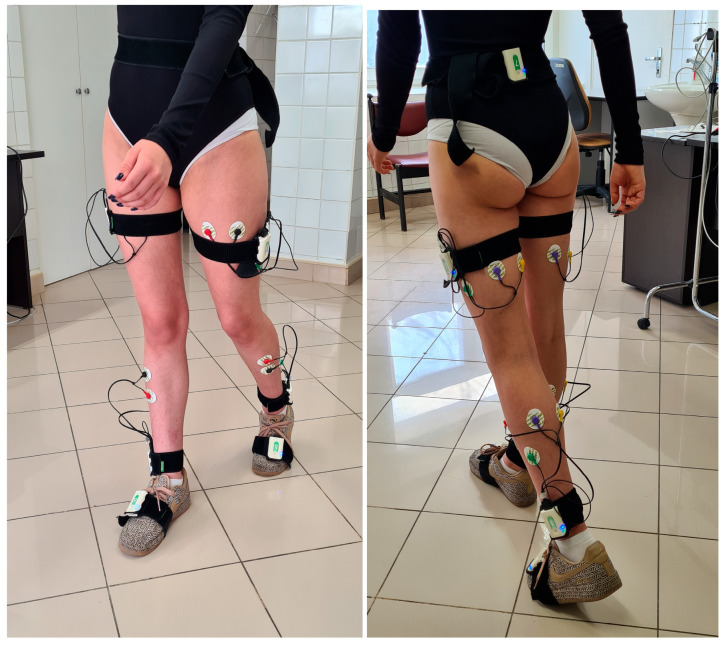
Gait analysis by IMU system and EMG electrode placement.

We used a modified technique for muscle electrode placement, differing from the SENIAM standard. We needed to obtain EMG from large muscle groups rather than individual muscles or their parts. Therefore, we placed electrodes transversely across the thigh in the middle third, both anteriorly (quadriceps and hamstrings). For the gastrocnemius muscle, one electrode was placed in the middle of the lateral head and the second in the middle of the medial head. Only the tibialis anterior muscle was recorded separately. This technique was used for the subsequent application of functional electrical stimulation technology to restore normal muscle automaticity (Figure 1).

The gait study was conducted at two speed levels: self-selected and fast. Both speed levels were self-reported. No additional measures were used.

The following temporal parameters were selected for analysis: gait cycle (GC) measured in seconds; stance phase (SP) measured as % of the GC; single stance phase (SS) measured as % of the GC; double stance phase (DS) measured as % of the GC; and the beginning of the terminal double limb stance phase (BTDLS). The BTDLS parameter reflects reciprocity. For each study, these parameters are presented as averages over 30 GCs.

The results of movement kinematics in the hip, knee, and ankle joints (flexion-extension), as well as in the muscles studied, are presented as an averaged graph over 30 GCs. Each graph contains a sequence of 200 values. For joint goniograms, the vertical axis is degrees, and the horizontal axis is % of the gait cycle. For the EMG profiles of muscle activity, the vertical axis is mV, and the horizontal axis is % of the gait cycle.

### 2.4. Statistical Analyses

Considering the nonlinear interactions between recovery time points, movement speed, and gait cycle phase classes, a balanced, asymmetric, full-factorial experimental design (FFE) of the 2 × 2 × 2 × 3 type with quantile partitioning of the functional curves was chosen. This approach allows for the identification of phase-dependent effects (e.g., quadriceps compensatory behavior vs. physiological normalization) inaccessible to traditional ANOVA or mixed models. Alternative methods (e.g., functional models) were excluded due to sample size limitations (*n* < 30) and the need to decompose higher-order interactions.

The analysis was conducted across two independent stages of the study, each with its own factor and dependent variable structure.

A 2 × 2 × 2 × 3 full-factorial design (rehabilitation time × walking speed × gait cycle phase × quantile classes) was chosen to decompose multivariate interactions with a limited sample size (*n* = 32). This design was justified by the following criteria.

Yates’ algorithm for orthogonal contrasts was used not for pairwise comparisons but for polynomial decomposition of the effects of the time and speed factors (linear/quadratic components). This allowed us to separate the overall effect into interpretable components (e.g., nonlinear recovery of knee range of motion 1 year after surgery), avoiding post-hoc corrections (Bonferroni), which, at *n* = 32, would have led to deflation of power (α_{adjusted} < 0.005). This strategy is in line with the recommendations of Maxwell et al. (2017) [22] for small-sample designs, where clinical interpretability is prioritized over maintaining strict significance.

Quantile partitioning (3 classes: 1–66%, 66–132%, 132–198% of the gait cycle) was used to localize phase-dependent anomalies (e.g., AMI of the quadriceps in the terminal phase of swing), which were not accessible when analyzing mean values. The alternative (uniform splitting by phase: stance/swing) was excluded because there was an autocorrelation problem, i.e., intra-phase points are not independent, which distorts the dispersion estimates in MANOVA. Regarding clinical motivations, peak deviations (e.g., 132–198% of the cycle) correlate with compensatory strategies (e.g., increased hip extension during fast walking after 1 year; d = 0.6). The sample limitation (*n* = 32) did not allow the use of functional methods (e.g., mixed models for curves), as there is a risk of overfitting: Higher-order polynomial components (*p* ≥ 3) gave unstable estimates (the standard error grew exponentially). In regards to the trade-off, FFE with orthogonal contrasts provides maximum power to detect interactions with limited degrees of freedom (df = 24–48), which is critical for phase-dependent effects (e.g., decreased quadriceps activity in the terminal phase after 0.5 years; *p* = 0.07; d = 0.45—a trend retained for clinical interpretation).

In regards to reproducibility testing, all contrasts were pre-specified and were not subject to data dredging.

Effects with *p* < 0.1 and d ≥ 0.4 are presented in the Appendix A for transparency (e.g., speed × time interaction for knee flexion at 1 year).

The sensitivity of the analysis was confirmed by simulation (*n* = 30, 1000 iterations): with d ≥ 0.5, the power reached 78% (at α = 0.05), corresponding to clinically significant effect sizes (e.g., amplitude recovery >10°).

The first stage of the study included dependent variables: GC, SP, SS, DS, and BTDLS. The factor space included four fixed factors: leg (2 levels: operated, healthy); speed (2 levels: self-selected, fast); subject (2 levels: healthy, operated); and time (3 levels: 0.5 year, 1 year, or more than 1 year of follow-up).

In regards to the rationale for factorial design and sample size, asymmetries in sample sizes (e.g., *n* = 12 vs. *n* = 14 for >1 year) are due to the clinical realities of long-term follow-up and do not compromise the balance of the FFE because all comparisons are carried out within the framework of multivariate criteria (Hotelling’s T^2^ and linear discriminant analysis (LDA)), where the weight of observations is determined by the informativeness of the predictors, and not by their number. The effective sample size for multivariate analyzes was adjusted using the Mahalanobis test (outlier exclusion > χ^2^_0.05_ (3)), resulting in *n* ≈ 12 while maintaining statistical power (1 − β > 0.8 for main effects). Within-subject correlations were accounted for through random intercepts in mixed models and paired *t*-tests with Holm–Bonferroni correction.

In regards to the quantile partitioning of curves, the division of goniograms/EMG into 3 phase classes (quantile intervals) is justified by the need to take into account the uneven distribution of biomechanical loads. Traditional phase divisions (heel-strike, stance phase, etc.) are not adapted to compensatory patterns. The quantile approach preserves the statistical independence of intraclass observations and allows the identification of local effects (for example, peak muscles inhibition of the musculus quadriceps femoris for terminal swing phase).

The use of the Hotelling test (T^2^) and LDA in the Appendix A is due to the need to clarify multivariate relationships with a limited sample and complex interaction structure:

The Hotelling test (T^2^) was used for the final verification of group differences in the predictor space (biomechanical and electrophysiological parameters) taking into account the correlational structure of the data, which provides additional analysis power compared to paired *t*-tests or MANOVA with small sample size.

The LDA method was used to visualize discriminant vectors following key factors (rehabilitation time, walking speed), identifying nonlinear cluster boundaries in asymmetric sample distributions. Furthermore, it was used to interpret the weighting coefficients of predictors in discriminant functions as indicators of clinically significant compensatory patterns inaccessible in linear models.

Both methods are not included in the main text, as their results serve as additional validation without adding any new interpretive value to the main findings. Their role is to confirm the stability of the findings when changing the analytical approach and to eliminate potential artifacts (e.g., multicollinearity in multivariate spaces).

The data analysis for the first stage was conducted using nonparametric methods after rank coding the dependent variables. The goal was to obtain accurate estimates of the significance of fixed factors and their interactions, without considering the coefficients of the study design’s orthogonal matrix.

In the second stage of the study, the dependent variables were goniogram parameters for the hip, knee, and ankle joints and bioelectrical activity profiles of the TA, GA, QA, HM muscles. The factor space included four fixed factors (Table 2).

**Figure 2:**
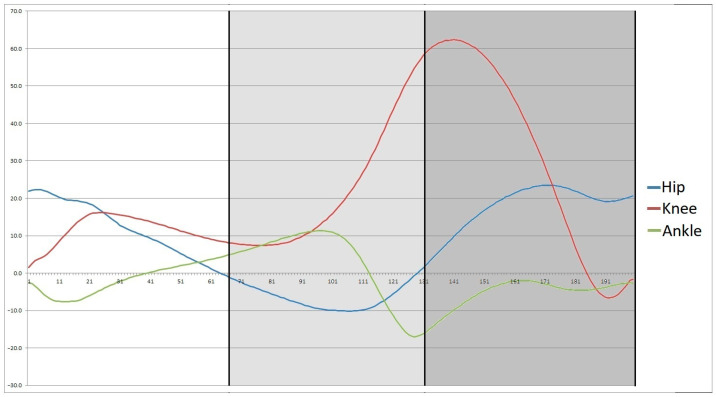
Dividing goniograms and muscle bioelectrical activity profiles into three intervals. Goniograms are presented as an example.

The division of goniograms and EMG profiles of muscles into three sections of the “class” factor is presented in Figure 2.

The “class” factor is a partition of the functional curve (goniogram or EMG) into three equally probable intervals (quantiles), based on the change in the curve’s pattern. To form the “class” factor, we used a quantile partitioning method: all 200 time points across all observations were combined into a single array, sorted in ascending order, and divided into three groups equal in number of observations—the 1st, 2nd, and 3rd quantiles (33.3%, 66.7%, and 100%). Thus, each “class” corresponds to a specific phase of the movement (initial, middle, and final), allowing investigation into how the influence of key factors (observation time, speed) on the biomechanical response varies depending on the phase of movement and thereby the identification of nonlinear and context-dependent effects that cannot be captured when analyzing average values across the entire goniogram or EMG profile.

Using quantile partitioning rather than uniform intervals ensures statistical balance and the correct application of orthogonal contrasts (linear and quadratic) for a factor with three levels. This approach is widely used in functional data analysis (FDA) [23] and enables a transition from a “point” to a “profile” interpretation of biomechanical changes.

Data are presented as b ± SE, where “b” is the effect estimate (slope) and SE is the standard error. A positive b value, or increase in the factor level, is associated with an increase in the response (e.g., a greater flexion angle with increasing load). A negative value of “b” indicates that an increase in the factor level is associated with a decrease in the response (e.g., a decrease in muscle activity with increasing load).

The experimental design for the second stage was a balanced, asymmetric 3 × 2 × 2 × 3 FFE. Statistical analysis utilized Yates’ method [24,25,26,27,28,29] for orthogonal contrasts. Yates’ method, an efficient computational algorithm for analyzing variance (ANOVA) and calculating multiple linear regression coefficients in balanced orthogonal designs, was used to analyze both designs. The purpose of the method was to calculate the significance (*p*-value) of the main effects of factors and their interactions and to assess the contribution of not only linear but also quadratic effects for three-level factors within a second-order polynomial model using orthogonal contrasts.

The orthogonal contrast system was defined as follows. For two-level factors (Leg, Speed, Patient), linear orthogonal contrast coefficients with values of −1 and +1 were used. For three-level factors (Time, Class), a system of orthogonal polynomials was used: linear contrast (L) (coefficients): −1, 0, +1; quadratic contrast (Q) (coefficients): +1, −2, +1.

Yates’ method allows for the calculation of regression coefficients for each orthogonal contrast. These coefficients are interpreted as slopes (weights) in the regression equation and are used to estimate the magnitude and direction of effects, as well as for prediction. In this study, the total number of observations across all cases at each level included 66 points. The goniogram and muscle activity profiles of the central tendencies of the studied samples allow for testing (Shapiro–Wilk W test and Levene’s test) for the applicability of parametric data analyses. If the normality (Shapiro–Wilk W test) and homoscedasticity (Levene’s test) tests were passed, parametric data analysis was used. Otherwise, the data were rank-coded and analyzed using nonparametric methods.

Where direct group parameters are given, they are presented as the median and 25th and 75th quartiles.

The level of statistical significance for all tests was set to *p* < 0.05. Both significant effects (*p* < 0.05) and trends (*p* < 0.1) were described.

In stage three of multivariate analysis, to apply Hotelling’s T^2^-test, which requires comparison of two independent groups, the multivariate factor space (2 × 2 × 2 × 3) was decomposed into a series of one-way comparisons. Each analysis focused on a single key factor (e.g., “group: healthy/affected limb”) while holding the remaining parameters (speed, time, movement mode) constant. Thus, each test was implemented as a two-level (2^1^) one-way experiment, ensuring the correct application of Hotelling’s T^2^ Test [30] to assess multivariate differences between groups. Therefore, data analysis was carried out using a one-way two-level experiment of the 21-type multivariate analysis, followed by LDA to assess the significance of the studied factors on the vector of dependent variables for the first and second stages of the study. The experimental model was a “gray box” model, enabling the establishment of relationships and weights for the dependent variables, combining them into a single vector for the object-parametric profile, and assessing profile variations under factor loadings. To that end, Hotelling’s T^2^ test was used as a special case of MANOVA.

## 3. Results

### 3.1. The First Stage of Analysis

Table 3 presents the walking time parameters at a self-selected speed for all groups.

Starting at 0.5 years, all parameters were within the range of the healthy control group. There were no significant asymmetries. A more in-depth analysis of these same parameters for two movement speed variants is provided in Table 4. The signs of the slopes and their *p*-values are shown for the dependent variables of the first stage of the study: 1—fixed factor “leg”; 2—fixed factor “speed”; 3—fixed factor “patient”; 4L—fixed factor “linear contrast time”; and 4Q—fixed factor “quadratic contrast time”.

For the GC, SP, SS, and DS parameters, no interactions were found between factors (all factors were fixed and independent). A response was found for the “speed” factor. A negative slope indicates that slower movement speed is associated with higher GC, SP, and DS values. For the SS parameter, a positive slope indicates that slower movement speed is associated with lower average SS values.

For the BTDLS parameter, no significant response was found for any fixed factors. However, significant between-factor interactions were identified: leg, patient, and observation time were interrelated (indicating a direct relationship between the factors).

To assess the combined effects of time since surgical reconstruction and gait speed on the multivariate profile (5-dimensional vector Y) of biomechanical parameters (including gait cycle, joint angles, and EMG amplitudes—five dimensions), Hotelling’s T^2^ test was used within a MANOVA approach. The results are presented in Table 5.

In self-selected walking at 0.5 years, a tendency towards difference was observed (T^2^ = 16.0, *p* = 0.06). The most pronounced multivariate differentiation (T^2^ = 21.5, *p* = 0.09) was observed in the group of patients at 1 year during fast walking. Although the value did not reach the strict level of statistical significance (*p* < 0.05), the effect was large (F_5.18_ = 2.86). In the over-1-year group and during fast walking in 0.5- and 1-year groups (except for the above), the T^2^ values did not exceed the threshold of 4.5, indicating the absence of significant multivariate dysfunction. Thus, dysfunction is reduced by > 1 year.

The interpretation of the LDA coefficients is presented in Table A1.

The discriminant function coefficients (B1–B5) reflect the contribution weights of each of the five parameters in forming a linear combination that best differentiates between the experimental conditions. The most informative patterns were found in the 1-year group with fast walking: GC, SP, SS and DS phases. These coefficients had extreme values, indicating a strong inverse correlation between the parameters; for example: a high weight of B2 (positive) corresponds to an increase in the hip joint angle during the stance phase, whereas B3 and B4 (negative) correspond to a decrease in the EMG amplitude of the assessed thigh muscles.

Table A2 shows the signs of the slopes and their *p*-values for the dependent variables of the second stage of the study. For all tables, fixed levels and their interactions were coded similarly: 1L—observation time linear contrast; 1Q—observation time quadratic contrast; 2—speed; 3—patient; 4L—linear contrast class; and 4Q—quadratic contrast class.

### 3.2. The Second Stage of Analysis

In the second stage of the analysis, a new factor, “class” (factor 4), was added to the model. A detailed analysis of the dependent variables revealed significant main effects (Table 4). For the hip joint, a complex effect was revealed. Class had a strong independent effect (both contrasts were significant). The main effects of time (quadratic trend) and numerous two- and three-factor interactions were also significant. Particularly important are the time-class interaction (1L4Q) and interactions with the “patient” factor (1Q3, 1L34Q, 34Q), indicating that hip dynamics depend on the GC period, the time since surgery, and the group (patients vs. healthy controls) and that these factors are interrelated. Therefore, the joint’s range of motion directly depended on the time since reconstruction.

A simpler pattern of effects was found for the KJ (Table 4). Significant independent effects were observed for GC period (linear trend), gait speed, and time since surgery (linear trend). The interactions did not reach statistical significance, although the two-way speed–GC period interaction demonstrated a clear trend. The negative coefficient for the speed factor indicated that an increase in speed was associated with a decrease in the KJ range of motion. The fast-walking test thus revealed residual dysfunction. Knee range of motion decreased with increasing walking speed in the 0.5- and 1-year-old groups. However, only an increase in range of motion is a normal physiological response.

For ankle function, both significant effects (*p* < 0.05) and trends (*p* < 0.1) were found. For the ankle, almost all main effects (class, patient, speed, time) were significant. A significant three-way time–speed–patient interaction (1Q23) was also found, demonstrating the complex interplay of these factors in influencing the ankle function index and gait speed.

Significant effects (*p* < 0.05) were found for the TA muscle, and the most complicated and complex pattern of influences was observed. All main effects were significant, as were numerous second- and third-order interactions (1Q3, 1L2, 24kQ, 1Q4L, 1Q24L, 34Q, 24L), indicating an extremely strong dependence of TA function on the combined levels of all studied factors.

For QA, the most complex pattern was revealed, with significant effects up to a four-factor interaction (1Q234Q). Nearly all factors and their combinations had a significant effect, indicating that quadriceps function was most sensitive to complex changes in experimental settings (time, speed, patients vs. healthy controls, class).

For the HM muscle, an extremely strong and complex effect of factors was revealed. The largest contributions were made by the main effects of class (4Q), speed (2), and the linear time trend (1L). The quadratic component of class (4Q) had the strongest positive effect. The linear time trend (1L) had the strongest negative effect, indicating a significant decrease in HM parameters over the observation period. Speed (2) had a strong positive effect. Thus, for the HM muscle, increasing walking speed normalizes its activity profile. Numerous significant two- and three-factor interactions were identified, particularly involving the time and class factors (1L4L, 1Q4L, 1L4Q, 24Q), highlighting the complex, interrelated nature of their effects on the HM muscle. The influence of the “patients vs. healthy controls” factor (3) was also significant and evident in interactions with other factors (Table A3).

In regards to the multivariate analysis of EMG responses (7-dimensional vector Y), in the second stage of the analysis, the vector was expanded to a 7-dimensional vector, including EMG amplitudes (tibialis anterior, gastrocnemius, quadriceps, hamstring). The results are presented in Table A4.

The T^2^ test results (Table A4) demonstrated a high statistical significance across all conditions. All T^2^ values are highly significant (*p* < 0.0001), indicating a strong, consistent influence of factors (time from surgery, speed of walking) on the electromyographic profile. The greatest multivariate dispersion (T^2^ = 1080) was observed during self-selected walking in the 0.5-year group, suggesting maximum neuromuscular instability during this rehabilitation period, even at low loads. During fast walking, T^2^ decreased from 1080 (0.5 years) to 47 (>1 year), reflecting a gradual normalization of neuromuscular activity over time and the beneficial effect of higher walking speed.

The most robust (least variable) profiles are in the >1-year group, with T^2^ ranging from 47 to 156, indicating the achievement of an adaptive equilibrium. The interpretation of LDA coefficients is given in Table A5.

Coefficients for joints and muscles (7 discriminant functions) show the contribution of each of the EMG parameters to the multidimensional profile. The most significant patterns included the following: in the 0.5-year group, slow walking, indicating the dominance of HA, TA, and GA activity with a decrease in QA activity. Therefore, the main stabilizer of the KJ—QA—cannot provide the necessary activity during this period, which is partially compensated for by other muscles, resulting in the overall decrease in load on the KJ.

At 1 year, for fast walking, all coefficients were close to zero, and no dominant muscles emerged due to the normalization of neuromuscular coordination, leading to the absence of dominant patterns. These findings are a key indicator of the restoration of automatism and motor control. Given the small number of patients, this result can be considered preliminary.

In the >1-year group, absolute values of the coefficients did not exceed 0.18, confirming the stability and recovery of neuromuscular synergy.

Minor deviations (e.g., B3 = –0.186 during self-selected walking) may reflect residual adaptations that do not affect functional efficiency.

Therefore, the electromyographic profile demonstrated a clear recovery pattern: from severe incoordination at 0.5 years (T^2^ = 1080, pronounced LDA patterns) to almost complete normalization by >1 year (T^2^ = 47–156, LDA coefficients close to zero), indicating successful restoration of neuromuscular control, especially during dynamic tasks (fast walking).

## 4. Discussion

Overall, the initial data analysis showed that gait function was almost completely restored by 0.5 years post-reconstruction, as evidenced by walking at a self-selected speed. None of the parameters changed significantly, remaining within the healthy range from 0.5 years onward. Thus, the greatest restoration of gait function occurs within 0.5 years after reconstruction. These findings are consistent with data from previous studies [31,32]. The mere fact of restoration of the GC temporal structure does not indicate complete recovery. More complex motor tasks cannot yet be performed during this period.

This study, for the first time, quantitatively assessed phase-dependent recovery of gait biomechanics 12 months after ACL reconstruction using Yates orthogonal contrasts (Table 2). The findings support the hypothesis of incomplete compensation of movements and highlight the need for an individualized approach to rehabilitation [31].

Our data confirmed the previously reported increase in the GC, SP, and DS parameters at lower speed [33]. A decrease in the SS parameter with decreasing speed was also observed, as this was the swing phase of the other leg. Accordingly, with an increase in SP, the swing phase time will decrease by the same amount. Therefore, the response to changes in gait speed in terms of time parameters, beginning 0.5 years after ACL reconstruction, was considered physiological. In the studied groups, walking reciprocity was not impaired (BTDLS parameter). A more detailed analysis revealed that all time-related parameters were independent. Moreover, even in the over 1-year group, complete recovery did not occur. The maximum differentiation of time-related parameters (according to the Hotelling T^2^ criterion) was observed in the 1-year group. These findings are consistent with our previous study [31] and the previously reported data [34]. However, whether complete recovery of gait function occurs at all and, if so, at what stage remains controversial across studies. According to a meta-analysis [12], most gait parameters during the stance period, including the maximum range of KJ flexion, did not differ significantly between individuals with ACL reconstruction and healthy controls. Only the range of extension in the middle of the stance period remained insufficient, but signs of improvement were observed with increasing time after reconstruction. Another study [35] confirmed that, 2 years after reconstruction, minor differences persisted on the operated side. Moreover, the semitendinosus muscle exhibits greater activity before the onset of the gait cycle, indicating its special role as an ACL agonist. The authors suggested that this injury is not only a mechanical lesion but also affects sensorimotor integrity.

Several studies reported complete recovery of gait biomechanics at a relatively early stage [12]. In contrast, others noted that some parameters did not recover [10,36]; these discrepancies may be related to differences in research methods, sensitivity, and resolution. While simple parameters such as gait speed or frequency recover quickly, abnormal amplitude-phase parameters of the joint itself or the lower limb muscles persist. However, they do not exert a functional or perceptible effect on the patient. The study suggested that the sensory deficit resulting from ACL loss affected reflex regulation of the KJ with no impact on normal walking. However, during peak strength and coordination loads, this regulation may be insufficient. Therefore, as discussed in this study, the operated joint remains more vulnerable to injury at any time after reconstruction. This hypothesis is supported by a study [32] that found muscle balance was not fully recovered within a year, which is consistent with our data.

In our study, movements in the operated KJ demonstrate several simple patterns. The first was a decrease in amplitude with increasing movement speed. In line with data from our previous study [36], this functional test clearly demonstrated that recovery was incomplete at this time. In the 0.5- and 1-year groups, the amplitude decreased by an average of 2 degrees as walking speed increased. Greater functional demands on the joint at a faster speed lead not to an increase in amplitude, but to a decrease, which was clearly evident by the decreased flexion amplitude during the swing period (Figure 2). The reason was obvious: the joint was not functionally prepared for higher loads. This effect diminished over time. For the over 1-year group, the amplitude no longer changed. It is better than a decrease, but the normal physiological response of an increase in amplitude was not reached. Moreover, an increase in the maximum flexion range during the swing period was observed across groups, ranging from 56 to 60 degrees. Therefore, there was a clear trend in the increase in KJ flexion range during the swing period over time. However, the pattern of changes in the range during the functional gait speed test never returned to normal, suggesting the development of changes in the injured joint that had a clear tendency to compensate over time, but did not fully recover. The joint remained less resilient to high loads.

The knee flexion range in the swing phase showed the greatest clinically significant decrease (Δ = 8.2°; ES = d = 0.60; 95% CI [−12.1°, −4.3°]; *p* < 0.001 (Table 3)). This deficit corresponds to arthrogenic rigidity [37] and requires correction as part of rehabilitation protocols after 6–12 months.

**Figure 3:**
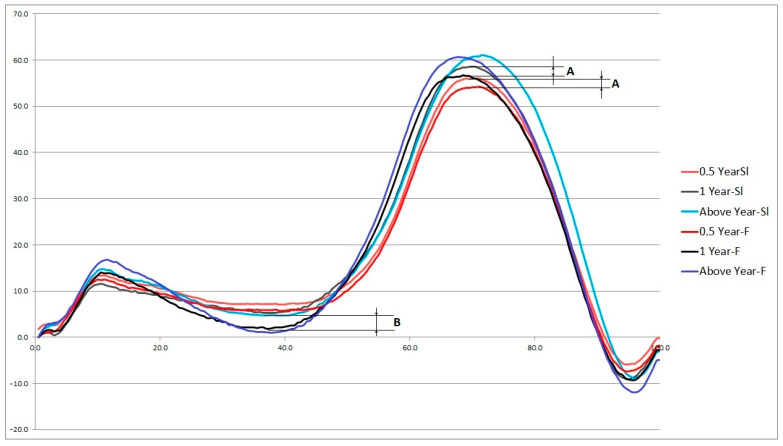
Average goniograms of movements in the operated KJ across all groups at self-selected and fast speeds. The vertical scale is degrees, and the horizontal scale is % GC. The “Sl” (slow) index is walking at a self-selected speed; the “F” (fast) index is walking at a fast speed. Amplitude “A” is a decrease in swing flexion during the transfer period by 2 degrees for the 0.5-YearSl and 1-YearSl groups, and amplitude “B” is an increase in the amplitude of extension in the middle of the support period at fast speed for the 1-Year and Over 1-Year groups.

Another characteristic change in hip function was an increase in hip extension amplitude during mid-stance, observed only in patients in the 1-year and >1-year groups. This increase in amplitude (recovery) occurred only during fast walking. At normal speed, this extension amplitude remained reduced (Figure 3).

Overall, the data from the first stage confirmed that gait function recovered from the 0.5-year group after reconstruction, where changes were greatest, to the least in the >1-year group. In this group, fast walking demonstrated the primary mechanism for maintaining high speed, which developed during the previous stages: an increase in hip joint range of motion with a simultaneous decrease in hip muscle EMG amplitude. We previously described this phenomenon [37]. Therefore, for most patients, fast walking was less asymmetrical (>0.5 years after reconstruction).

At 0.5 years, the quadriceps femoris remained the most severely affected muscle. In the 0.5-year group, 0.5 years, recovery occurred in both its function and that of the other muscles studied. However, this could only be concluded for gait function, which is relevant for sports activities involving peak loads. At a high level, coordination remained insufficient, which may lead to recurrent injuries. Phase-dependent m. quadriceps activation (Table 4, p. B) shows a moderate effect (f^2^ = 0.22, *p* = 0.005) when transitioning from slow to fast walking, which explains patients’ complaints of fatigue [12]. Yates contrasts revealed a significant Time × Speed interaction (Table A2), supporting the need for dynamic testing in rehabilitation.

The data also showed that the most gait recovery occurred within three months after reconstruction [35,38,39].

For walking at a self-selected speed at 0.5 years after reconstruction, compensatory responses can be realized through changes across the entire gait cycle. Therefore, despite the completely normal gait parameters in this group, recovery was incomplete, and active adaptation persisted. Moreover, walking at a higher speed showed a better degree of compensation. This is quite consistent with the data of the study by Lee H, et al. [40] who found a smaller contribution of the KJ to the energy of the operated lower limb during walking compared to the contralateral one. In the 1-year-after-reconstruction group, normalization of residual biomechanical changes occurred specifically during fast walking. A higher speed offset these changes, confirming our previous finding that, despite virtually normal walking parameters by year one, recovery was not complete [31]. A 2026 study by Esmaeili A. et al. [41] shows that the reduction KJ amplitude during gait persists at one year. The authors also note that minor deviations in gait biomechanics are persistent but inconsistent across studies. Brisk walking also demonstrated better functional performance during this period, with a faster walking speed than that of healthy individuals, as previously described in the long-term period [37]. These changes create a specific pattern of dysfunction, characterized by greater hip joint amplitudes and decreased thigh muscle activity. We have previously observed this effect [37] during the residual period of 3 years or more after reconstruction. The data analysis method enabled the detection of this pattern at earlier stages. This adaptation mechanism apparently developed within one year after reconstruction and became permanent. Moreover, at 1 year post-surgery, during fast walking, the most pronounced multivariate imbalance in biomechanical parameters was observed (although it did not reach statistical significance at *p* < 0.05), indicating a latent but clinically significant dysfunction that was not manifested in individual parameters but became apparent only when the overall profile was analyzed. The running exercise test used by Ge X et al. [42] in patients who underwent surgery nine months earlier revealed asymmetries in quadriceps strength and biomechanical abnormalities in the knee and hip joints. This indirectly confirms the patterns we discovered but for a more demanding movement test—running. Thus, fast walking can partially compensate for existing changes in the biomechanics of movement. This study found a clear effect of normalization (or a tendency towards it) of many biomechanical parameters during fast walking. However, the extent to which it can be used for rehabilitation purposes remains to be seen.

For the >1-year group, no multivariate dysfunction was detected, which can be considered an indirect sign of the completion of functional restructuring processes.

In the second stage of data analysis, the pattern of influences became significantly more complex. While in the first stage, for most indicators (except for BTDLS), the influences were relatively simple (main effects), in the second stage, significant interactions, often of high order, were identified for most variables (joints and muscles). The most complex, non-additive influence was observed for parameters related to muscles and large muscle groups (TA, QA, HM).

The absence of significant effects for higher-order interactions (e.g., Time × Speed × Phase; *p* = 0.08; Table A4) suggests a predominance of linear compensatory strategies over complex adaptations. These data are excluded from the main discussion but are available for detailed analysis (Table A3).

This study confirms the research hypothesis. Complete restoration of gait biomechanics did not occur. These results highlight the impossibility of reducing the analysis to the study of isolated effects. Understanding biomechanics requires considering the combined influence of various parameters. Yates’ orthogonal contrasts (methodology, p. X) allowed us to differentiate clinically significant effects from statistical noise. A focus on phase-dependent rehabilitation (Table A1, Table A2, Table A3, Table A4 and Table A5) is recommended for personalized protocols. Furthermore, we consider this work preliminary. Data collection is ongoing.

Limitations of the study include a relatively small sample size (*n* < 30 per group). An additional limitation was that not all patients could be consistently assessed at all three timepoints. Some patients changed their place of residence or were lost to follow-up. Despite a common post-surgical rehabilitation protocol, not all patients followed it strictly. Furthermore, there were individual differences in the recovery period that could not be avoided or standardized. Furthermore, heterogeneity is introduced by the fact that patients were admitted for examination from two different clinics and underwent surgery, albeit using the same technique, but by different surgeons. This also introduces heterogeneity into the primary study data. Due to their professional backgrounds, all patients undergoing surgery spent the year following reconstruction with varying levels of physical activity, adhering to key necessary restrictions. Except that, we lacked the technical capability to study patients’ gait biomechanics immediately before surgery. Some patients were tested at various times before surgery. However, only a small number were tested within a few days of the surgery.

Further research could include machine learning to classify statuses (“healthy,” “compensatory,” “at risk of relapse”) based on these discriminant functions, which would be the next logical step in advancing digital medical rehabilitation. The question of the required ACL implant tension also remains open, which inevitably affects the biomechanics of the joint. Both the ACL and posterior cruciate ligaments significantly increase the extension and flexion gap [43].

## 5. Conclusions

In the 0.5-years-after-ACL-reconstruction group, gait function is restored sufficiently for the patient to walk at a voluntary speed. However, knee and hip muscle function remains poor. In groups 1-year and more-than-a-year, biomechanical indicators are better. Therefore, the main medical rehabilitation measures should be implemented within the first six months after surgery. In the group 1-year-after-ACL-reconstruction, full restoration of KJ function and walking did not occur, but all the main recovery processes were complete. However, this can only be determined by instrumental methods. Specifically, as walking speed increased, the KJ range of motion decreased (not a physiological response pattern). In the over-1-year group, this decrease did not occur, but there was also no physiological increase in the range of motion. Moreover, the group from 0.5-years to over-1-year, KJ’s range of motion gradually increased. Overall, the KJ remained less resistant to increased physical activity for up to 1 year after reconstruction.

The recovery process involved all major muscle groups. The primary target was the quadriceps femoris. The primary mechanism of adaptation to high loads (speed) was characterized by increased hip range of motion and decreased EMG amplitude in the thigh muscles. Fast walking also reduced functional asymmetry between the operated and non-operated legs. Fast walking may be useful as a functional assessment condition to reveal residual asymmetry and load-dependent compensatory strategies.

To enable these results to be used for clinical decision making, larger longitudinal studies with standardized rehabilitation and repeated follow-up of each patient are needed.

## Figures and Tables

**Table 1:** Characteristics of patient groups after ACL reconstruction. Note: number—number of patients; age—age in years; height—height in centimeters; BMI—body mass index.

Group	Men				Women			
	Number	Age	Height	BMI	Number	Age	Height	BMI
0.5 year	9	36.4	179.3	29.0	3	31.3	163.6	22.3
1 year	6	39.1	178.1	29.8	1	22	160.0	20.3
>1 year	9	34.3	181.7	28.2	4	32	163.2	24.1

**Table 2:** Factors and levels.

Factor	Number of Levels	List of Levels
Observation time	3	0.5-year 1-year >1-year
Speed	2	Self-selected Fast
Subject	2	Operated Healthy
Class	3	1–66 points; 66–132 points; 132–198 points

**Table 3:** Walking time parameters at a self-selected speed.

Temporal Parameters	Side	0.5-Year	1-Year	>1-Year	Healthy Group (*n* = 34)
GC (s)	Affected	1.2 [1.1:1.3]	1.1 [1.1:1.2]	1.2 [1.1:1.2]	1.1 [1.1; 1.2]
	Non-affected	1.2 [1.1:1.3]	1.1 [1.1:1.2]	1.2 [1.1:1.2]	
SP (%)	Affected	63.2 [62.7:64:0]	63.4 [62.1:64:7]	63.6 [62.8:65.1]	63.1 [62.4; 64.4]
	Non-affected	64.2 [63.0:65:8]	63.8 [63.8:63.8]	64.2 [62.9:65.0]	
SS (%)	Affected	35.8 [33.8:36.4]	36.0 [35.4:37.3]	36.1 [35.0:36.8]	36.9 [35.7; 37.9]
	Non-affected	36.8 [35.8:37.1]	36.4 [35.6:38.1]	36.4 [35.2:37.3]	
DS (%)	Affected	27.7 [26.4:30.2]	27.2 [24.8:29.5]	27.8 [25.529.5]	26.1 [24.6; 28.1]
	Non-affected	27.4 [26.4:30.5]	27.4 [25.0:29.4]	27.9 [25.5:29.8]	
BTDLS (%)	Affected	49.5 [49.1:50.2]	49.6 [49.5:50.2]	50.2 [49.8:50.6]	49.9 [49.6; 50.3]
	Non-affected	50.3 [49.7:50.7]	50.4 [49.6:50.9]	50.0 [49.6:50.6]	

Where ‘s’ it is measured in seconds, the other parameters are in % of the duration ot the GC.

**Table 4:** First stage of analysis (temporal characteristics of the gait cycle).

Factor	GC		SP		SS		DS		BTDLS	
	b Sign	*p*-Value	b Sign	*p*-Value	b Sign	*p*-Value	b Sign	*p*-Value	b Sign	*p*-Value
1234Q	−	0.940	+	0.807	+	0.944	−	0.519	−	0.090
1234L	−	0.895	+	0.990	−	0.700	−	0.374	+	0.105
234Q	+	0.940	+	0.414	+	0.708	+	0.502	−	0.887
234L	+	0.895	−	0.672	−	0.855	+	0.315	−	0.805
134Q	+	0.940	+	0.439	+	0.416	+	0.573	+	0.044
134L	+	0.895	−	0.062	−	0.159	+	0.294	−	0.017
124Q	+	0.940	−	0.923	+	0.552	+	0.749	+	0.669
124L	+	0.895	+	0.617	−	0.871	+	0.152	+	1.000
123	−	0.915	+	0.616	+	0.901	−	0.469	+	0.055
34Q	−	0.940	+	0.750	+	0.833	−	0.620	−	0.569
34L	−	0.895	+	0.084	+	0.195	−	0.294	+	0.006
24Q	−	0.497	−	0.579	+	0.409	−	0.210	+	0.118
24L	+	0.362	+	0.672	−	0.808	−	0.467	+	0.579
23		0.915	+	0.850	+	0.722	+	0.397	−	0.467
14Q	−	0.705	−	0.994	−	0.916	−	0.528	−	0.722
14L	+	0.157	+	0.292	−	0.262	−	1.000	−	0.711
13		0.915	+	0.706	+	0.927	+	0.315	−	0.033
12	−	0.593	−	0.300	+	0.291	−	0.821	+	0.940
4Q	+	0.120	−	0.888	+	0.734	+	0.812	+	0.803
4L	−	0.157	−	0.768	+	0.839	+	0.399	−	0.758
3	−	0.915	−	0.309	−	0.337	−	0.377	−	0.111
2	−	0.000	−	0.000	+	0.000	−	0.000	+	0.706
1	−	0.244	−	0.081	+	0.180	−	0.020	+	0.633

In the table above, the “+” and “−” signs indicate the direction of the effects of the slope coefficients of the multiple polynomial regression used in the modified version of the Yates method for assessing trends in the dependent variable.

**Table 5:** Statistical indicators of the multivariate profile for the 5-dimensional analysis of dependent variables (GC, SP, SS, DS, HB BTDLS), obtained using Hotelling’s T^2^ test.

Time	Speed	T^2^	F_5,18_	*p*-Value
0.5-year	Self-selected	16.0	2.62	0.06
	Fast	3.96	0.65	0.67
1-year	Self-selected	4.30	0.57	0.72
	Fast	21.5	2.86	0.09
>1-year	Self-selected	4.00	0.65	0.66
	Fast	4.00	0.65	0.66

## Data Availability

The data obtained during the study are available in the open Mendeley Data repository (DOI: 10.17632/xt3kzt59vr.1)—https://data.mendeley.com/datasets/xt3kzt59vr/1 (accessed on 11 February 2026).

## Abbreviations

The following abbreviations are used in this manuscript:

ACL	anterior cruciate ligament
AMI	arthrogenic muscle inhibition
ANOVA	analysis of variance
BMI	body mass index
BTDLS	beginning of the terminal double limb stance phase
CP	control profile
DO	distal offset
DS	double stance phase
EMG	electromyography
FEA	fractional experimental analysis
FFE	full-factorial experimental design
GC	gait cycle
GM	gastrocnemius muscle
HM	hamstring muscle
IMU	inertial measurement unit
KJ	knee joint
LDA	linear discriminant analysis
MANOVA	multivariate analysis of variance
OO	overall offset
QA	quadriceps muscle
QF	quadriceps femoris
RTS	return to sport
SE	standard error
SP	stance phase
SS	single stance phase
TA	tibialis anterior
T^2^	Hotelling’s T-squared statistic

## Appendix A

**Table A1:** Coefficients obtained using LDA for the 5-dimensional, dependent-variable analysis stage (GC, SP, SS, DS, BTDLS).

Time	Speed	B1	B2	B3	B4	B5
0.5-year	Self-selected	1.29	10.82	−8.76	−9.79	0.66
	Fast	0.448	−0.702	−0.459	0.091	0.348
1-year	Self-selected	2.074	−0.472	−2.505	−1.143	0.454
	Fast	−15.13	71.73	−81.65	−75.50	−2.07
>1-year	Self-selected	−1.464	0.405	−1.398	−0.903	1.121
	Fast	5.092	−8.499	8.057	8.296	−0.737

Note: B1—GC; B2—SP; B3—SS; B4—DS; B5—BTDLS.

**Table A2:** Signs of the slopes and their *p*-values for the dependent variables of the second stage of the study. Where “b” is the effect estimate (slope), SE is the standard error.

Factor	Hip		Knee		Ankle		TA		GA		QA		HM	
	b ± SE	*p*-Value	b ± SE	*p*-Value	b ± SE	*p*-Value	b ± SE	*p*-Value	b ± SE	*p*-Value	b ± SE	*p*-Value	b ± SE	*p*-Value
1234Q	0.024 ± 0.065	0.715	0.033 ± 0.192	0.863	−0.014 ± 0.068	0.841	−0.030 ± 0.491	0.952	−0.090 ± 0.596	0.880	**0.743** ± **0.203**	**0.000**	0.018 ± 0.306	0.954
1234L	−0.082 ± 0.112	0.462	0.064 ± 0.332	0.848	−0.070 ± 0.117	0.550	−0.167 ± 0.851	0.844	−0.216 ± 1.032	0.834	−0.674 ± 0.352	0.055	−0.402 ± 0.530	0.449
234Q	−0.056 ± 0.112	0.615	−0.017 ± 0.332	0.959	−0.107 ± 0.117	0.359	−0.251 ± 0.851	0.768	−0.605 ± 1.032	0.558	0.158 ± 0.352	0.653	0.008 ± 0.530	0.988
234L	0.025 ± 0.194	0.898	0.275 ± 0.575	0.633	−0.082 ± 0.203	0.686	−1.787 ± 1.474	0.225	0.848 ± 1.788	0.635	−0.372 ± 0.609	0.541	−0.254 ± 0.918	0.782
134Q	−0.023 ± 0.091	0.803	−0.033 ± 0.271	0.904	0.043 ± 0.096	0.652	0.131 ± 0.695	0.850	−1.143 ± 0.843	0.175	**−0.619** ± **0.287**	**0.031**	−0.941 ± 0.433	0.030
134L	−0.014 ± 0.158	0.932	−0.134 ± 0.470	0.776	−0.141 ± 0.166	0.396	−0.237 ± 1.204	0.844	0.814 ± 1.459	0.577	0.701 ± 0.497	0.159	0.867 ± 0.750	0.248
124Q	**0.113** ± **0.065**	**0.079**	−0.054 ± 0.192	0.779	0.059 ± 0.068	0.383	−0.165 ± 0.491	0.737	−0.284 ± 0.596	0.634	**1.385** ± **0.203**	**0.000**	−0.113 ± 0.306	0.711
124L	−0.055 ± 0.112	0.622	−0.107 ± 0.332	0.747	0.022 ± 0.117	0.850	0.977 ± 0.851	0.251	**−2.951** ± **1.032**	**0.004**	**−1.113** ± **0.352**	**0.002**	−0.977 ± 0.530	0.066
123	−0.004 ± 0.065	0.946	−0.146 ± 0.192	0.448	0.007 ± 0.068	0.915	−0.154 ± 0.491	0.753	−0.336 ± 0.596	0.572	−0.236 ± 0.203	0.245	−1.045 ± 0.306	0.001
34Q	−0.017 ± 0.112	0.880	−0.114 ± 0.332	0.731	0.115 ± 0.117	0.325	**1.853** ± **0.851**	**0.030**	0.155 ± 1.032	0.881	0.006 ± 0.352	0.985	−0.023 ± 0.530	0.965
34L	0.045 ± 0.091	0.624	0.045 ± 0.271	0.869	−0.277 ± 0.096	0.004	0.657 ± 0.695	0.345	−0.447 ± 0.843	0.596	0.443 ± 0.287	0.123	−0.532 ± 0.433	0.219
24Q	**−0.263** ± **0.112**	**0.019**	−0.325 ± 0.332	0.328	0.024 ± 0.117	0.840	−0.539 ± 0.851	0.527	0.413 ± 1.032	0.689	**1.198** ± **0.352**	**0.001**	−0.637 ± 0.530	0.229
24L	−0.005 ± 0.194	0.981	−0.327 ± 0.575	0.569	0.030 ± 0.203	0.882	−1.130 ± 1.474	0.443	1.244 ± 1.788	0.486	**−1.790** ± **0.609**	**0.003**	−1.658 ± 0.918	0.071
23	0.036 ± 0.112	0.749	0.010 ± 0.332	0.975	0.189 ± 0.117	0.107	0.462 ± 0.851	0.587	−0.015 ± 1.032	0.988	0.097 ± 0.352	0.782	−1.117 ± 0.530	0.035
14Q	−0.101 ± 0.194	0.604	0.042 ± 0.575	0.942	0.130 ± 0.203	0.523	0.198 ± 1.474	0.893	−1.293 ± 1.788	0.470	**2.330** ± **0.609**	**0.000**	0.869 ± 0.918	0.344
14L	−0.189 ± 0.158	0.232	0.046 ± 0.470	0.922	0.226 ± 0.166	0.173	0.273 ± 1.204	0.820	−1.203 ± 1.459	0.410	−0.600 ± 0.497	0.228	1.219 ± 0.750	0.104
13	**0.182** ± **0.091**	**0.047**	0.249 ± 0.271	0.358	−0.128 ± 0.096	0.179	**1.566** ± **0.695**	**0.024**	**−3.740** ± **0.843**	**0.000**	**−0.618** ± **0.287**	**0.031**	−0.486 ± 0.433	0.262
12	0.066 ± 0.158	0.675	0.219 ± 0.470	0.642	−0.318 ± 0.166	0.054	−0.774 ± 1.204	0.520	−1.465 ± 1.459	0.315	−0.222 ± 0.497	0.655	2.327 ± 0.750	0.002
4Q	**0.573** ± **0.091**	**0.000**	−0.507 ± 0.271	0.062	0.877 ± 0.096	0.000	**2.369** ± **0.695**	**0.001**	**−4.816** ± **0.843**	**0.000**	−0.288 ± 0.287	0.316	5.342 ± 0.433	0.000
4L	0.183 ± 0.158	0.247	−0.875 ± 0.470	0.063	0.038 ± 0.166	0.819	**−2.730** ± **1.204**	**0.023**	**−8.325** ± **1.459**	**0.000**	**−4.530** ± **0.497**	**0.000**	2.045 ± 0.750	0.006
3	**−0.271** ± **0.129**	**0.036**	0.014 ± 0.383	0.972	−0.244 ± 0.135	0.071	1.099 ± 0.983	0.263	−0.474 ± 1.192	0.691	0.002 ± 0.406	0.997	−1.307 ± 0.612	0.033
2	**0.127** ± **0.065**	**0.050**	0.082 ± 0.192	0.669	−0.005 ± 0.068	0.945	0.600 ± 0.491	0.222	0.039 ± 0.596	0.947	**−0.461** ± **0.203**	**0.023**	−0.391 ± 0.306	0.201
1	0.005 ± 0.112	0.967	−0.187 ± 0.332	0.574	0.073 ± 0.117	0.533	**2.478** ± **0.851**	**0.004**	0.464 ± 1.032	0.653	−0.213 ± 0.352	0.546	−2.313 ± 0.530	0.000
1234Q	**−0.410** ± **0.091**	**0.000**	−0.149 ± 0.271	0.582	−0.131 ± 0.096	0.172	**3.333** ± **0.695**	**0.000**	0.015 ± 0.843	0.985	2.694 ± 0.287	0.000	−2.571 ± 0.433	0.000
1234L	0.048 ± 0.091	0.600	−0.233 ± 0.271	0.391	0.016 ± 0.096	0.868	0.336 ± 0.695	0.628	0.711 ± 0.843	0.399	−0.511 ± 0.287	0.075	−1.693 ± 0.433	0.000
234Q	**0.310** ± **0.112**	**0.006**	−0.060 ± 0.332	0.856	−0.006 ± 0.117	0.961	0.965 ± 0.851	0.257	1.291 ± 1.032	0.211	0.535 ± 0.352	0.128	−2.482 ± 0.530	0.000
234L	0.091 ± 0.194	0.638	0.517 ± 0.575	0.368	−0.115 ± 0.203	0.572	2.362 ± 1.474	0.109	1.006 ± 1.788	0.574	**4.270** ± **0.609**	**0.000**	6.404 ± 0.918	0.000
134Q	0.190 ± 0.158	0.230	0.024 ± 0.470	0.958	−0.250 ± 0.166	0.132	−0.411 ± 1.204	0.733	**−7.713** ± **1.459**	**0.000**	**−3.284** ± **0.497**	**0.000**	4.070 ± 0.750	0.000
134L	**0.706** ± **0.158**	**0.000**	0.770 ± 0.470	0.101	0.306 ± 0.166	0.065	**3.646** ± **1.204**	**0.002**	1.832 ± 1.459	0.210	**−5.229** ± **0.497**	**0.000**	−4.073 ± 0.750	0.000
124Q	**7.610** ± **0.091**	**0.000**	−0.453 ± 0.271	0.095	**−1.276** ± **0.096**	**0.000**	**18.127** ± **0.695**	**0.000**	**−24.743** ± **0.843**	**0.000**	**3.957** ± **0.287**	**0.000**	**21.077** ± **0.433**	**0.000**
124L	**4.111** ± **0.158**	**0.000**	**9.973** ± **0.470**	**0.000**	**−1.234** ± **0.166**	**0.000**	**13.120** ± **1.204**	**0.000**	**−39.101** ± **1.459**	**0.000**	**−13.934** ± **0.497**	**0.000**	**2.521** ± **0.750**	**0.001**
123	0.078 ± 0.129	0.545	0.401 ± 0.383	0.296	**0.281** ± **0.135**	**0.038**	**4.221** ± **0.983**	**0.000**	**3.476** ± **1.192**	**0.004**	**5.304** ± **0.406**	**0.000**	**−2.289** ± **0.612**	**0.000**
34Q	0.042 ± 0.129	0.746	**−0.903** ± **0.383**	**0.019**	**−0.433** ± **0.135**	**0.001**	**28.797** ± **0.983**	**0.000**	**19.543** ± **1.192**	**0.000**	**20.992** ± **0.406**	**0.000**	**19.350** ± **0.612**	**0.000**
34L	**0.325** ± **0.091**	**0.000**	−0.144 ± 0.271	0.596	**1.034** ± **0.096**	**0.000**	**2.922** ± **0.695**	**0.000**	−0.854 ± 0.843	0.311	−0.323 ± 0.287	0.260	**−2.661** ± **0.433**	**0.000**
24Q	−0.143 ± 0.158	0.365	**1.494** ± **0.470**	**0.001**	**1.162** ± **0.166**	**0.000**	**9.452** ± **1.204**	**0.000**	**5.014** ± **1.459**	**0.001**	**−15.506** ± **0.497**	**0.000**	**−11.792** ± **0.750**	**0.000**

Significant effects are highlighted in bold, and trends are underlined.

**Table A3:** Coefficients obtained using LDA for the 5-dimensional, dependent-variable analysis stage (GC, SP, SS, DS, BTDLS).

Dependent Variable	Main Effects	Significant Interactions (Order > 1)	Nature of Effects
Hip	4L + 8.1, 4Q + 7.9, 1Q − 2.4	1Q3, 1L34Q, 24Q, 34Q	Strong, primarily class × time
Knee	4L + 5.9, 2 + 3.8, 1L − 1.9	no (trend. 24Q/24L *p* = 0.08)	Additive
Ankle	4L + 6.8, 4Q + 4.7, 3 − 2.0, 2 + 4.1, 1Q − 1.7	1Q23	All factors were significant; the key interaction was “time × speed × patient.”
“TA”	4L + 9.2, 4Q + 6.1, 3 − 2.5, 2 + 3.9, 1L − 2.8, 1Q − 1.4	1Q3, 1L2, 24Q, 1Q24L, 34Q, 24L + 4 etc.	Maximum non-additive, “confusion” of all factors
“GA”	4L + 6.3, 4κQ + 5.4, 3 − 1.8, 2 + 4.5, 1L − 2.1	34Q, 24Q, 24L, 1Q34L	Strong class and speed, moderate 3-factor relationships
“QA”	4L + 5.5, 4Q + 6.0, 3 − 2.2, 2 + 3.7, 1L − 1.9	15 interaction, including 1Q234L	Nonseparable, 4-factor interference
“HM”	4Q + 21.1, 2 + 19.4, 1L − 11.8, 3 − 2.3	1Q24Q, 1L4Q, 24L, 34Q, 23 etc.	Strongest “class × time” and “speed × time”; overall η^2^ = 0.78

**Table A4:** Coefficients obtained using LDA for the 5-dimensional, dependent-variable analysis stage (GC, SP, SS, DS, BTDLS).

Time	Speed	T^2^	F_7,388_	*p*-Value
0.5-year	Self-selected	1080	152	<0.0001
	Fast	550	77	<0.0001
1-year	Self-selected	167	23	<0.0001
	Fast	65	9	<0.0001
>1-year	Self-selected	156	22	<0.0001
	Fast	47	7	<0.0001

**Table A5:** Values of coefficients B1-B7 obtained using LDA for the 5-dimensional study stage of dependent variables (for joints: hip, knee, ankle; muscles: TA, GA, QA, HM).

Time	Speed	B1	B2	B3	B4	B5	B6	B7
0.5-year	Self-selected	−0.956	0.272	−0.241	0.010	0.003	−0.328	0.415
	Fast	−0.639	0.251	0.222	0.026	0.002	−0.088	0.191
1-year	Self-selected	0.053	−0.199	−0.087	0.061	−0.037	−0.092	−0.133
	Fast	0.168	−0.146	−0.133	−0.001	−0.014	−0.004	−0.063
>1-year	Self-selected	−0.112	−0.010	−0.186	−0.025	0.002	−0.118	0.088
	Fast	−0.036	0.050	−0.042	−0.022	0.007	0.006	0.051

Note: B1—Hip; B2—Knee; B3—Ankle; B4—TA; B5—GA; B6—QA; B7—HM.
